# Supplementary material for: Ecological niche modeling the potential geographic distribution of four Culicoides species of veterinary significance in Florida, USA
Source: PLoS One. 2019 Feb 15;14(2):e0206648. doi: 10.1371/journal.pone.0206648 (PMC6377124; doi:10.1371/journal.pone.0206648)
Supplement: S1 Table — Coordinates of each presence point used to build the final models for each of the four species used in this study: C. insignis, C. stellifer, C. debilipalpis, and C. venustus. These coordinates are adjusted from the original dataset to remove duplicates in the same raster. As such the dataset is spatially unique at 1.0km. (DOCX) [file pone.0206648.s001.docx]

| *Culicoides* Species | Latitude | Longitude |
| --- | --- | --- |
| *C. insignis* | 26.69208 | -81.59857 |
| *C. insignis* | 26.69785 | -81.42214 |
| *C. insignis* | 27.20739 | -82.40958 |
| *C. insignis* | 26.93669 | -81.32620 |
| *C. insignis* | 26.95232 | -81.69711 |
| *C. insignis* | 26.94595 | -81.69621 |
| *C. insignis* | 26.94862 | -81.69680 |
| *C. insignis* | 27.10727 | -81.80623 |
| *C. insignis* | 27.67909 | -82.34827 |
| *C. insignis* | 27.67587 | -82.35483 |
| *C. insignis* | 27.07967 | -82.04282 |
| *C. insignis* | 27.07433 | -82.04561 |
| *C. insignis* | 27.07852 | -82.03360 |
| *C. insignis* | 27.07350 | -82.05738 |
| *C. insignis* | 27.08252 | -82.02407 |
| *C. insignis* | 27.07294 | -82.03603 |
| *C. insignis* | 27.06928 | -82.05741 |
| *C. insignis* | 27.07480 | -82.03274 |
| *C. insignis* | 27.08042 | -82.02401 |
| *C. insignis* | 27.07504 | -82.03730 |
| *C. insignis* | 27.07610 | -82.04592 |
| *C. insignis* | 27.07588 | -82.04212 |
| *C. insignis* | 27.07511 | -82.04636 |
| *C. insignis* | 27.07608 | -82.05712 |
| *C. insignis* | 27.07643 | -82.04602 |
| *C. insignis* | 27.07797 | -82.04784 |
| *C. insignis* | 26.40468 | -81.13325 |
| *C. insignis* | 26.99309 | -81.96311 |
| *C. insignis* | 26.98958 | -81.95983 |
| *C. insignis* | 25.50531 | -81.12888 |
| *C. insignis* | 26.40999 | -81.14806 |
| *C. insignis* | 25.50405 | -81.12542 |
| *C. insignis* | 26.41577 | -81.14736 |
| *C. insignis* | 26.41584 | -81.14275 |
| *C. insignis* | 26.41071 | -81.12948 |
| *C. insignis* | 26.40683 | -81.13959 |
| *C. insignis* | 26.40996 | -81.14643 |
| *C. insignis* | 26.40589 | -81.13637 |
| *C. insignis* | 26.40666 | -81.13206 |
| *C. insignis* | 26.40841 | -81.12704 |
| *C. insignis* | 26.41068 | -81.14988 |
| *C. insignis* | 26.40881 | -81.14506 |
| *C. insignis* | 26.40822 | -81.14198 |
| *C. insignis* | 27.62278 | -81.81323 |
| *C. insignis* | 27.62058 | -81.80523 |
| *C. insignis* | 27.61832 | -81.80816 |
| *C. insignis* | 27.42931 | -82.32015 |
| *C. insignis* | 27.43069 | -82.31852 |
| *C. insignis* | 27.42931 | -82.32015 |
| *C. insignis* | 27.42931 | -82.32015 |
| *C. insignis* | 27.43069 | -82.31852 |
| *C. insignis* | 27.43069 | -82.31852 |
| *C. insignis* | 27.42931 | -82.32015 |
| *C. insignis* | 26.72689 | -81.10924 |
| *C. insignis* | 26.38162 | -81.01828 |
| *C. insignis* | 26.38940 | -81.00150 |
| *C. insignis* | 26.39067 | -81.00204 |
| *C. insignis* | 26.38131 | -81.01708 |
| *C. insignis* | 26.39123 | -81.00655 |
| *C. insignis* | 26.38067 | -81.01735 |
| *C. insignis* | 26.73171 | -81.90239 |
| *C. insignis* | 26.73194 | -81.89301 |
| *C. insignis* | 26.68019 | -81.08342 |
| *C. insignis* | 27.09139 | -82.29340 |
| *C. insignis* | 27.08698 | -82.29609 |
| *C. insignis* | 27.08967 | -82.29042 |
| *C. insignis* | 26.97256 | -81.73673 |
| *C. insignis* | 26.97359 | -81.74241 |
| *C. insignis* | 26.97271 | -81.73945 |
| *C. insignis* | 26.97107 | -81.73887 |
| *C. insignis* | 27.42931 | -82.32015 |
| *C. insignis* | 27.43069 | -82.31852 |
| *C. insignis* | 27.44490 | -80.60598 |
| *C. insignis* | 27.44486 | -80.59633 |
| *C. insignis* | 27.42652 | -80.60242 |
| *C. insignis* | 27.42728 | -80.61000 |
| *C. insignis* | 27.43079 | -80.61104 |
| *C. insignis* | 27.32042 | -81.84583 |
| *C. insignis* | 27.31895 | -81.84453 |
| *C. insignis* | 27.32724 | -81.85065 |
| *C. insignis* | 27.32877 | -81.84965 |
| *C. insignis* | 27.43495 | -80.58744 |
| *C. insignis* | 27.43881 | -80.58866 |
| *C. insignis* | 26.54927 | -81.78451 |
| *C. insignis* | 26.55034 | -81.78013 |
| *C. insignis* | 26.71054 | -81.79295 |
| *C. insignis* | 26.08369 | -81.54262 |
| *C. insignis* | 26.06215 | -81.54221 |
| *C. insignis* | 26.01101 | -81.53780 |
| *C. insignis* | 26.05102 | -81.53436 |
| *C. insignis* | 26.78932 | -81.54799 |
| *C. insignis* | 26.78915 | -81.54810 |
| *C. insignis* | 26.78819 | -81.54843 |
| *C. insignis* | 26.78826 | -81.54899 |
| *C. insignis* | 26.78880 | -81.54891 |
| *C. insignis* | 26.78881 | -81.54818 |
| *C. insignis* | 26.78808 | -81.54830 |
| *C. insignis* | 26.78826 | -81.54826 |
| *C. insignis* | 26.78857 | -81.54871 |
| *C. insignis* | 27.15242 | -81.75130 |
| *C. insignis* | 27.15459 | -81.75273 |
| *C. insignis* | 27.15473 | -81.75509 |
| *C. insignis* | 27.16338 | -81.75125 |
| *C. insignis* | 27.17320 | -81.75015 |
| *C. insignis* | 27.17603 | -81.74788 |
| *C. insignis* | 27.17794 | -81.74426 |
| *C. insignis* | 27.18069 | -81.74328 |
| *C. insignis* | 27.17969 | -81.74714 |
| *C. insignis* | 27.17971 | -81.74885 |
| *C. insignis* | 27.43056 | -82.32163 |
| *C. insignis* | 27.42791 | -82.32137 |
| *C. insignis* | 27.42791 | -82.32137 |
| *C. insignis* | 27.43056 | -82.32163 |
| *C. insignis* | 27.43056 | -82.32163 |
| *C. insignis* | 27.42791 | -82.32137 |
| *C. insignis* | 27.78141 | -82.13999 |
| *C. insignis* | 27.77560 | -82.14120 |
| *C. insignis* | 27.76686 | -82.14135 |
| *C. insignis* | 27.77660 | -82.14515 |
| *C. insignis* | 27.76914 | -82.14013 |
| *C. insignis* | 27.14261 | -82.08166 |
| *C. insignis* | 27.14326 | -82.08423 |
| *C. insignis* | 27.14465 | -82.08421 |
| *C. insignis* | 27.14465 | -82.08671 |
| *C. insignis* | 27.14614 | -82.08939 |
| *C. insignis* | 27.14126 | -82.07004 |
| *C. insignis* | 27.14269 | -82.06659 |
| *C. insignis* | 27.14497 | -82.06345 |
| *C. insignis* | 27.14848 | -82.06244 |
| *C. insignis* | 27.13130 | -82.09192 |
| *C. insignis* | 27.13051 | -82.08541 |
| *C. insignis* | 27.12597 | -82.08526 |
| *C. insignis* | 27.13422 | -82.07813 |
| *C. insignis* | 27.15177 | -82.09871 |
| *C. insignis* | 27.15150 | -82.09728 |
| *C. insignis* | 27.14958 | -82.09293 |
| *C. insignis* | 27.13440 | -82.09628 |
| *C. insignis* | 27.13458 | -82.09929 |
| *C. insignis* | 27.13119 | -82.08702 |
| *C. insignis* | 27.13000 | -82.08143 |
| *C. insignis* | 27.13711 | -82.09118 |
| *C. insignis* | 27.14952 | -82.10650 |
| *C. insignis* | 27.14748 | -82.10653 |
| *C. insignis* | 27.14335 | -82.10654 |
| *C. insignis* | 27.14116 | -82.10652 |
| *C. insignis* | 29.16419 | -81.16493 |
| *C. insignis* | 29.16525 | -81.18989 |
| *C. insignis* | 29.20060 | -81.16850 |
| *C. insignis* | 29.16024 | -81.18849 |
| *C. insignis* | 26.73610 | -81.19681 |
| *C. insignis* | 26.73604 | -81.19308 |
| *C. insignis* | 26.70896 | -81.17791 |
| *C. insignis* | 27.43069 | -82.31852 |
| *C. insignis* | 27.43069 | -82.31852 |
| *C. insignis* | 27.42931 | -82.32015 |
| *C. insignis* | 27.42931 | -82.32015 |
| *C. insignis* | 27.42931 | -82.32015 |
| *C. insignis* | 26.79970 | -81.95082 |
| *C. insignis* | 28.36891 | -82.11688 |
| *C. insignis* | 28.37490 | -82.11444 |
| *C. insignis* | 28.38618 | -82.10263 |
| *C. insignis* | 27.46236 | -81.51853 |
| *C. insignis* | 27.46294 | -81.51914 |
| *C. insignis* | 27.46221 | -81.51701 |
| *C. insignis* | 27.46931 | -81.51580 |
| *C. insignis* | 26.99644 | -82.27959 |
| *C. insignis* | 26.99273 | -82.27002 |
| *C. insignis* | 26.99148 | -82.26719 |
| *C. insignis* | 27.42931 | -82.32015 |
| *C. insignis* | 27.42931 | -82.32015 |
| *C. insignis* | 27.42931 | -82.32015 |
| *C. insignis* | 27.42931 | -82.32015 |
| *C. insignis* | 27.43069 | -82.31852 |
| *C. insignis* | 27.43069 | -82.31852 |
| *C. insignis* | 27.42931 | -82.32015 |
| *C. insignis* | 27.42931 | -82.32015 |
| *C. insignis* | 27.43069 | -82.31852 |
| *C. insignis* | 27.43069 | -82.31852 |
| *C. insignis* | 26.57435 | -81.32713 |
| *C. insignis* | 26.58094 | -81.30019 |
| *C. insignis* | 26.56660 | -81.29416 |
| *C. insignis* | 26.49577 | -81.29287 |
| *C. insignis* | 26.50646 | -81.27862 |
| *C. insignis* | 27.43069 | -82.31852 |
| *C. insignis* | 27.43069 | -82.31852 |
| *C. insignis* | 27.43069 | -82.31852 |
| *C. insignis* | 27.43069 | -82.31852 |
| *C. insignis* | 27.42931 | -82.32015 |
| *C. insignis* | 27.42931 | -82.32015 |
| *C. insignis* | 27.43069 | -82.31852 |
| *C. insignis* | 27.43069 | -82.31852 |
| *C. insignis* | 27.43069 | -82.31852 |
| *C. insignis* | 27.42931 | -82.32015 |
| *C. insignis* | 27.42931 | -82.32015 |
| *C. insignis* | 27.42931 | -82.32015 |
| *C. insignis* | 27.96884 | -81.47247 |
| *C. insignis* | 27.96915 | -81.45399 |
| *C. insignis* | 27.96907 | -81.44780 |
| *C. insignis* | 27.99129 | -81.48554 |
| *C. insignis* | 27.42931 | -82.32015 |
| *C. insignis* | 27.42931 | -82.32015 |
| *C. insignis* | 27.43069 | -82.31852 |
| *C. insignis* | 27.43069 | -82.31852 |
| *C. insignis* | 27.43069 | -82.31852 |
| *C. insignis* | 27.42931 | -82.32015 |
| *C. insignis* | 27.42931 | -82.32015 |
| *C. insignis* | 27.42931 | -82.32015 |
| *C. insignis* | 27.43069 | -82.31852 |
| *C. insignis* | 27.43069 | -82.31852 |
| *C. insignis* | 27.43069 | -82.31852 |
| *C. insignis* | 27.58300 | -81.02289 |
| *C. insignis* | 29.91343 | -82.57833 |
| *C. insignis* | 29.91362 | -82.57907 |
| *C. insignis* | 29.91533 | -82.57980 |
| *C. insignis* | 29.91551 | -82.58071 |
| *C. insignis* | 29.91555 | -82.58161 |
| *C. insignis* | 29.91550 | -82.58196 |
| *C. insignis* | 29.91792 | -82.58566 |
| *C. insignis* | 29.91959 | -82.59009 |
| *C. insignis* | 29.71517 | -82.46162 |
| *C. insignis* | 29.71386 | -82.46135 |
| *C. insignis* | 29.71386 | -82.45996 |
| *C. insignis* | 29.71360 | -82.46043 |
| *C. insignis* | 29.71264 | -82.46046 |
| *C. insignis* | 28.09814 | -81.56521 |
| *C. insignis* | 28.09869 | -81.56480 |
| *C. insignis* | 28.09970 | -81.56423 |
| *C. insignis* | 28.09966 | -81.56344 |
| *C. insignis* | 28.10475 | -81.56288 |
| *C. insignis* | 28.10557 | -81.56388 |
| *C. insignis* | 28.10145 | -81.56402 |
| *C. insignis* | 28.51659 | -81.87972 |
| *C. insignis* | 27.42931 | -82.32015 |
| *C. insignis* | 27.42931 | -82.32015 |
| *C. insignis* | 27.42931 | -82.32015 |
| *C. insignis* | 27.43069 | -82.31852 |
| *C. insignis* | 27.43069 | -82.31852 |
| *C. insignis* | 27.43069 | -82.31852 |
| *C. insignis* | 27.43069 | -82.31852 |
| *C. insignis* | 27.42931 | -82.32015 |
| *C. insignis* | 27.42931 | -82.32015 |
| *C. insignis* | 27.42931 | -82.32015 |
| *C. insignis* | 27.43069 | -82.31852 |
| *C. insignis* | 27.43069 | -82.31852 |
| *C. insignis* | 26.69058 | -81.86747 |
| *C. insignis* | 26.86534 | -81.71229 |
| *C. insignis* | 26.86614 | -81.71618 |
| *C. insignis* | 26.87565 | -81.71786 |
| *C. insignis* | 26.69674 | -81.65249 |
| *C. insignis* | 26.32420 | -80.19747 |
| *C. insignis* | 26.75903 | -81.80186 |
| *C. insignis* | 27.43069 | -82.31852 |
| *C. insignis* | 27.43069 | -82.31852 |
| *C. insignis* | 27.42931 | -82.32015 |
| *C. insignis* | 27.42931 | -82.32015 |
| *C. insignis* | 27.42931 | -82.32015 |
| *C. insignis* | 27.42931 | -82.32015 |
| *C. insignis* | 27.42931 | -82.32015 |
| *C. insignis* | 27.43069 | -82.31852 |
| *C. insignis* | 27.43069 | -82.31852 |
| *C. insignis* | 27.42931 | -82.32015 |
| *C. insignis* | 27.42931 | -82.32015 |
| *C. insignis* | 27.42931 | -82.32015 |
| *C. insignis* | 26.30521 | -81.78751 |
| *C. insignis* | 26.30521 | -81.78751 |
| *C. insignis* | 26.29590 | -81.79198 |
| *C. insignis* | 27.24512 | -82.27372 |
| *C. insignis* | 27.24258 | -82.26165 |
| *C. insignis* | 27.26007 | -82.28737 |
| *C. insignis* | 27.26696 | -82.28460 |
| *C. insignis* | 27.27124 | -82.27832 |
| *C. insignis* | 27.27320 | -82.27167 |
| *C. insignis* | 27.28073 | -82.26118 |
| *C. insignis* | 26.70596 | -81.18616 |
| *C. insignis* | 26.71786 | -81.17772 |
| *C. insignis* | 26.71021 | -81.19428 |
| *C. insignis* | 26.72119 | -81.18710 |
| *C. insignis* | 26.70886 | -81.17985 |
| *C. insignis* | 26.70538 | -81.18581 |
| *C. insignis* | 26.71012 | -81.17908 |
| *C. insignis* | 26.71456 | -81.18047 |
| *C. insignis* | 27.43069 | -82.31852 |
| *C. insignis* | 27.43069 | -82.31852 |
| *C. insignis* | 27.42931 | -82.32015 |
| *C. insignis* | 27.42931 | -82.32015 |
| *C. insignis* | 26.98768 | -81.75010 |
| *C. insignis* | 26.97110 | -81.73869 |
| *C. insignis* | 26.97274 | -81.74155 |
| *C. insignis* | 26.97162 | -81.73845 |
| *C. insignis* | 26.96770 | -81.73601 |
| *C. insignis* | 26.69339 | -81.59850 |
| *C. insignis* | 26.68904 | -81.59813 |
| *C. insignis* | 26.69072 | -81.59376 |
| *C. insignis* | 26.69259 | -81.59674 |
| *C. insignis* | 26.49428 | -81.17634 |
| *C. insignis* | 26.50207 | -81.18977 |
| *C. insignis* | 26.50381 | -81.18951 |
| *C. insignis* | 26.50930 | -81.19862 |
| *C. insignis* | 26.50846 | -81.20328 |
| *C. insignis* | 26.50862 | -81.21123 |
| *C. insignis* | 26.50890 | -81.21432 |
| *C. insignis* | 26.50844 | -81.21739 |
| *C. insignis* | 26.51095 | -81.22434 |
| *C. insignis* | 26.48904 | -81.17610 |
| *C. insignis* | 26.49024 | -81.17605 |
| *C. insignis* | 26.50500 | -81.20756 |
| *C. insignis* | 26.49939 | -81.20731 |
| *C. insignis* | 26.49489 | -81.20707 |
| *C. insignis* | 26.48850 | -81.20679 |
| *C. insignis* | 26.48681 | -81.21143 |
| *C. insignis* | 26.46844 | -81.18990 |
| *C. insignis* | 26.45731 | -81.18959 |
| *C. insignis* | 26.45097 | -81.18975 |
| *C. insignis* | 26.47009 | -81.18830 |
| *C. insignis* | 26.47182 | -81.17653 |
| *C. insignis* | 26.96189 | -80.30261 |
| *C. insignis* | 26.97379 | -80.28871 |
| *C. insignis* | 26.97705 | -80.29518 |
| *C. insignis* | 26.98060 | -80.31353 |
| *C. insignis* | 26.75840 | -81.97322 |
| *C. insignis* | 26.74148 | -81.97975 |
| *C. insignis* | 26.74099 | -81.98915 |
| *C. insignis* | 26.79219 | -81.98970 |
| *C. insignis* | 26.80408 | -81.98773 |
| *C. insignis* | 26.76703 | -81.97257 |
| *C. insignis* | 26.79392 | -81.99040 |
| *C. insignis* | 26.79834 | -81.98817 |
| *C. insignis* | 26.79894 | -81.98796 |
| *C. insignis* | 27.43066 | -82.32156 |
| *C. insignis* | 27.43066 | -82.32156 |
| *C. insignis* | 27.42931 | -82.32015 |
| *C. insignis* | 27.42931 | -82.32015 |
| *C. insignis* | 27.42931 | -82.32015 |
| *C. insignis* | 27.42931 | -82.32015 |
| *C. insignis* | 27.43066 | -82.32156 |
| *C. insignis* | 27.43066 | -82.32156 |
| *C. insignis* | 27.43066 | -82.32156 |
| *C. insignis* | 27.43066 | -82.32156 |
| *C. insignis* | 27.43066 | -82.32156 |
| *C. insignis* | 27.42931 | -82.32015 |
| *C. insignis* | 27.42931 | -82.32015 |
| *C. insignis* | 27.42931 | -82.32015 |
| *C. insignis* | 26.27373 | -81.82746 |
| *C. insignis* | 27.28741 | -80.95773 |
| *C. insignis* | 27.28751 | -80.95153 |
| *C. insignis* | 27.28725 | -80.94843 |
| *C. insignis* | 27.28741 | -80.94496 |
| *C. insignis* | 27.28532 | -80.94488 |
| *C. insignis* | 27.27483 | -80.94467 |
| *C. insignis* | 27.27285 | -80.94745 |
| *C. insignis* | 27.27662 | -80.95210 |
| *C. insignis* | 27.27288 | -80.94139 |
| *C. insignis* | 27.27293 | -80.94479 |
| *C. insignis* | 27.27277 | -80.95620 |
| *C. insignis* | 27.27662 | -80.95917 |
| *C. insignis* | 26.37336 | -81.60617 |
| *C. insignis* | 26.37358 | -81.60585 |
| *C. insignis* | 26.37377 | -81.60568 |
| *C. insignis* | 26.37442 | -81.60519 |
| *C. insignis* | 26.40091 | -81.58679 |
| *C. insignis* | 26.40125 | -81.58465 |
| *C. insignis* | 26.38261 | -81.60304 |
| *C. insignis* | 26.37734 | -81.60377 |
| *C. insignis* | 26.53022 | -81.46658 |
| *C. insignis* | 26.45085 | -81.49177 |
| *C. insignis* | 26.45872 | -81.49438 |
| *C. insignis* | 26.46049 | -81.49503 |
| *C. insignis* | 26.46439 | -81.49447 |
| *C. insignis* | 26.45616 | -81.49405 |
| *C. insignis* | 27.13524 | -82.28658 |
| *C. insignis* | 27.13523 | -82.27282 |
| *C. insignis* | 27.13530 | -82.25513 |
| *C. insignis* | 27.15680 | -82.28964 |
| *C. insignis* | 27.16342 | -82.31356 |
| *C. insignis* | 27.14621 | -82.25418 |
| *C. insignis* | 27.14739 | -82.28603 |
| *C. insignis* | 28.71062 | -81.48595 |
| *C. insignis* | 28.71155 | -81.46346 |
| *C. insignis* | 28.71026 | -81.45956 |
| *C. insignis* | 28.72707 | -81.48881 |
| *C. insignis* | 28.72760 | -81.48177 |
| *C. insignis* | 28.75072 | -81.48216 |
| *C. insignis* | 27.43066 | -82.32156 |
| *C. insignis* | 27.43066 | -82.32156 |
| *C. insignis* | 27.42931 | -82.32015 |
| *C. insignis* | 27.42931 | -82.32015 |
| *C. insignis* | 27.42931 | -82.32015 |
| *C. insignis* | 27.42931 | -82.32015 |
| *C. insignis* | 27.43066 | -82.32156 |
| *C. insignis* | 27.43066 | -82.32156 |
| *C. insignis* | 27.43066 | -82.32156 |
| *C. insignis* | 27.42931 | -82.32015 |
| *C. insignis* | 27.42931 | -82.32015 |
| *C. insignis* | 27.42931 | -82.32015 |
| *C. insignis* | 27.42931 | -82.32015 |
| *C. insignis* | 27.42931 | -82.32015 |
| *C. insignis* | 26.45502 | -81.72223 |
| *C. insignis* | 26.45488 | -81.71959 |
| *C. insignis* | 26.45869 | -81.71929 |
| *C. insignis* | 26.84765 | -82.03551 |
| *C. insignis* | 27.43069 | -82.31852 |
| *C. insignis* | 27.43069 | -82.31852 |
| *C. insignis* | 27.43069 | -82.31852 |
| *C. insignis* | 27.42931 | -82.32015 |
| *C. insignis* | 27.42931 | -82.32015 |
| *C. insignis* | 27.42931 | -82.32015 |
| *C. insignis* | 27.42931 | -82.32015 |
| *C. insignis* | 27.43069 | -82.31852 |
| *C. insignis* | 27.43069 | -82.31852 |
| *C. insignis* | 27.42931 | -82.32015 |
| *C. insignis* | 27.42931 | -82.32015 |
| *C. insignis* | 27.42931 | -82.32015 |
| *C. insignis* | 27.42931 | -82.32015 |
| *C. insignis* | 26.63559 | -81.42336 |
| *C. insignis* | 26.63942 | -81.42886 |
| *C. insignis* | 26.63051 | -81.42867 |
| *C. insignis* | 26.62448 | -81.42836 |
| *C. insignis* | 26.61957 | -81.42583 |
| *C. insignis* | 26.61338 | -81.42358 |
| *C. insignis* | 26.61215 | -81.41994 |
| *C. insignis* | 26.60962 | -81.41677 |
| *C. insignis* | 26.60496 | -81.41626 |
| *C. insignis* | 26.59971 | -81.41688 |
| *C. insignis* | 26.61695 | -81.40380 |
| *C. insignis* | 26.63137 | -81.41783 |
| *C. insignis* | 26.62277 | -81.40609 |
| *C. insignis* | 26.59719 | -81.40537 |
| *C. insignis* | 26.63651 | -81.42375 |
| *C. insignis* | 26.63418 | -81.42103 |
| *C. insignis* | 26.63040 | -81.41626 |
| *C. insignis* | 26.63020 | -81.41045 |
| *C. insignis* | 27.43069 | -82.31852 |
| *C. insignis* | 27.43069 | -82.31852 |
| *C. insignis* | 27.42931 | -82.32015 |
| *C. insignis* | 27.42931 | -82.32015 |
| *C. insignis* | 27.42931 | -82.32015 |
| *C. insignis* | 27.42931 | -82.32015 |
| *C. insignis* | 27.42931 | -82.32015 |
| *C. insignis* | 27.43069 | -82.31852 |
| *C. insignis* | 27.43069 | -82.31852 |
| *C. insignis* | 27.42931 | -82.32015 |
| *C. insignis* | 27.42931 | -82.32015 |
| *C. insignis* | 27.42931 | -82.32015 |
| *C. insignis* | 27.42931 | -82.32015 |
| *C. insignis* | 27.42931 | -82.32015 |
| *C. insignis* | 27.02565 | -81.87536 |
| *C. insignis* | 27.02960 | -81.87352 |
| *C. insignis* | 27.02644 | -81.87681 |
| *C. insignis* | 27.02475 | -81.88285 |
| *C. insignis* | 27.02036 | -81.88530 |
| *C. insignis* | 27.01795 | -81.88645 |
| *C. insignis* | 27.01254 | -81.89038 |
| *C. insignis* | 27.00500 | -81.87540 |
| *C. insignis* | 27.01357 | -81.87690 |
| *C. insignis* | 27.00586 | -81.87673 |
| *C. insignis* | 27.00976 | -81.87727 |
| *C. insignis* | 27.00982 | -81.87610 |
| *C. insignis* | 27.00516 | -81.87419 |
| *C. insignis* | 27.00505 | -81.87691 |
| *C. insignis* | 27.01018 | -81.87814 |
| *C. insignis* | 27.01245 | -81.87667 |
| *C. insignis* | 27.01680 | -81.87492 |
| *C. insignis* | 29.96319 | -81.61470 |
| *C. insignis* | 29.95650 | -81.61984 |
| *C. insignis* | 29.95547 | -81.63077 |
| *C. insignis* | 29.96031 | -81.63206 |
| *C. insignis* | 29.97241 | -81.63813 |
| *C. insignis* | 29.96715 | -81.64626 |
| *C. insignis* | 29.96128 | -81.65510 |
| *C. insignis* | 29.95308 | -81.64348 |
| *C. insignis* | 30.05914 | -81.84330 |
| *C. insignis* | 30.05588 | -81.83955 |
| *C. insignis* | 30.06035 | -81.83481 |
| *C. insignis* | 30.06651 | -81.83130 |
| *C. insignis* | 30.06939 | -81.83844 |
| *C. insignis* | 30.08008 | -81.83943 |
| *C. insignis* | 30.12975 | -81.57612 |
| *C. insignis* | 27.19832 | -82.37294 |
| *C. insignis* | 27.20320 | -82.37592 |
| *C. insignis* | 27.20303 | -82.35841 |
| *C. insignis* | 27.18672 | -82.37286 |
| *C. insignis* | 27.17439 | -82.37226 |
| *C. insignis* | 27.17433 | -82.36897 |
| *C. insignis* | 27.17440 | -82.36424 |
| *C. insignis* | 27.18150 | -82.37221 |
| *C. insignis* | 27.20861 | -82.39828 |
| *C. insignis* | 27.18877 | -82.40571 |
| *C. insignis* | 28.80770 | -82.30546 |
| *C. insignis* | 28.51660 | -82.57587 |
| *C. insignis* | 27.64600 | -82.14224 |
| *C. insignis* | 27.64566 | -82.14128 |
| *C. insignis* | 27.64502 | -82.14143 |
| *C. insignis* | 27.64492 | -82.14241 |
| *C. insignis* | 27.64496 | -82.14365 |
| *C. insignis* | 27.64606 | -82.14298 |
| *C. insignis* | 27.43069 | -82.31852 |
| *C. insignis* | 27.43069 | -82.31852 |
| *C. insignis* | 27.43069 | -82.31852 |
| *C. insignis* | 27.42931 | -82.32015 |
| *C. insignis* | 27.42931 | -82.32015 |
| *C. insignis* | 27.42931 | -82.32015 |
| *C. insignis* | 27.42931 | -82.32015 |
| *C. insignis* | 28.09853 | -81.56471 |
| *C. insignis* | 28.09967 | -81.56425 |
| *C. insignis* | 28.09970 | -81.56345 |
| *C. insignis* | 28.10583 | -81.56251 |
| *C. insignis* | 28.09841 | -81.56532 |
| *C. insignis* | 28.09843 | -81.56569 |
| *C. insignis* | 28.09820 | -81.56525 |
| *C. insignis* | 28.09862 | -81.56543 |
| *C. insignis* | 27.19699 | -81.91871 |
| *C. insignis* | 27.19800 | -81.91823 |
| *C. insignis* | 27.19594 | -81.92016 |
| *C. insignis* | 27.19554 | -81.91810 |
| *C. insignis* | 28.51653 | -81.87967 |
| *C. insignis* | 28.51717 | -81.87992 |
| *C. insignis* | 28.51680 | -81.88056 |
| *C. insignis* | 28.51528 | -81.88047 |
| *C. insignis* | 28.51534 | -81.87915 |
| *C. insignis* | 30.23495 | -83.71829 |
| *C. insignis* | 29.75030 | -83.34534 |
| *C. insignis* | 27.67722 | -81.44847 |
| *C. insignis* | 27.67375 | -81.44256 |
| *C. insignis* | 27.66964 | -81.43591 |
| *C. insignis* | 27.65845 | -81.39404 |
| *C. insignis* | 27.67926 | -81.41325 |
| *C. insignis* | 27.70540 | -81.44648 |
| *C. insignis* | 27.78402 | -81.46033 |
| *C. insignis* | 27.78336 | -81.45531 |
| *C. insignis* | 27.78753 | -81.44866 |
| *C. insignis* | 27.78578 | -81.44905 |
| *C. insignis* | 27.78172 | -81.44844 |
| *C. insignis* | 27.78005 | -81.44835 |
| *C. insignis* | 27.77798 | -81.44766 |
| *C. insignis* | 27.77649 | -81.44716 |
| *C. insignis* | 29.57193 | -81.64622 |
| *C. insignis* | 29.57203 | -81.64816 |
| *C. insignis* | 29.57169 | -81.64969 |
| *C. insignis* | 29.57161 | -81.65132 |
| *C. insignis* | 29.57084 | -81.65129 |
| *C. insignis* | 29.56927 | -81.65144 |
| *C. insignis* | 29.57132 | -81.65561 |
| *C. insignis* | 29.57009 | -81.65561 |
| *C. insignis* | 29.57567 | -81.58917 |
| *C. insignis* | 29.57157 | -81.58580 |
| *C. insignis* | 29.57192 | -82.72590 |
| *C. insignis* | 29.56460 | -81.55664 |
| *C. insignis* | 27.43069 | -82.31852 |
| *C. insignis* | 27.43069 | -82.31852 |
| *C. insignis* | 27.43069 | -82.31852 |
| *C. insignis* | 27.42931 | -82.32015 |
| *C. insignis* | 27.42931 | -82.32015 |
| *C. insignis* | 27.42931 | -82.32015 |
| *C. insignis* | 27.42931 | -82.32015 |
| *C. insignis* | 27.42931 | -82.32015 |
| *C. insignis* | 27.43069 | -82.31852 |
| *C. insignis* | 27.43069 | -82.31852 |
| *C. insignis* | 27.43069 | -82.31852 |
| *C. insignis* | 27.43069 | -82.31852 |
| *C. insignis* | 27.42931 | -82.32015 |
| *C. insignis* | 27.42931 | -82.32015 |
| *C. insignis* | 27.42931 | -82.32015 |
| *C. insignis* | 27.42931 | -82.32015 |
| *C. insignis* | 27.42931 | -82.32015 |
| *C. insignis* | 29.14290 | -81.48405 |
| *C. insignis* | 29.14643 | -81.46805 |
| *C. insignis* | 29.14275 | -81.44836 |
| *C. insignis* | 29.16286 | -81.46393 |
| *C. insignis* | 29.18079 | -81.49280 |
| *C. insignis* | 29.17993 | -81.49540 |
| *C. insignis* | 29.17856 | -81.49554 |
| *C. insignis* | 29.17462 | -81.50320 |
| *C. insignis* | 28.69362 | -81.12413 |
| *C. insignis* | 28.69185 | -81.12362 |
| *C. insignis* | 28.69050 | -81.12127 |
| *C. insignis* | 28.68915 | -81.11781 |
| *C. insignis* | 28.71014 | -81.06086 |
| *C. insignis* | 28.71114 | -81.05565 |
| *C. insignis* | 28.71228 | -81.04866 |
| *C. insignis* | 29.11972 | -81.53093 |
| *C. insignis* | 29.10358 | -81.54536 |
| *C. insignis* | 29.08297 | -81.56145 |
| *C. insignis* | 29.09151 | -81.62805 |
| *C. insignis* | 29.08105 | -81.52012 |
| *C. insignis* | 26.62597 | -81.74138 |
| *C. insignis* | 26.62597 | -81.74138 |
| *C. insignis* | 26.64390 | -81.73376 |
| *C. insignis* | 26.64390 | -81.73376 |
| *C. insignis* | 26.64989 | -81.73949 |
| *C. insignis* | 26.64391 | -81.73222 |
| *C. insignis* | 26.64391 | -81.73222 |
| *C. insignis* | 26.64337 | -81.72906 |
| *C. insignis* | 26.64126 | -81.73987 |
| *C. insignis* | 26.64064 | -81.74134 |
| *C. insignis* | 26.64014 | -81.73732 |
| *C. insignis* | 26.64026 | -81.73899 |
| *C. insignis* | 27.09270 | -81.82710 |
| *C. insignis* | 27.09270 | -81.82710 |
| *C. insignis* | 27.10196 | -81.81164 |
| *C. insignis* | 27.10599 | -81.81686 |
| *C. insignis* | 27.10715 | -81.80932 |
| *C. insignis* | 27.10567 | -81.81273 |
| *C. insignis* | 27.10567 | -81.81273 |
| *C. insignis* | 27.10729 | -81.80626 |
| *C. insignis* | 27.09268 | -81.81217 |
| *C. insignis* | 27.09268 | -81.81217 |
| *C. insignis* | 25.99317 | -81.59505 |
| *C. insignis* | 26.00569 | -81.57579 |
| *C. insignis* | 26.00569 | -81.57579 |
| *C. insignis* | 25.99990 | -81.57842 |
| *C. insignis* | 26.00200 | -81.57138 |
| *C. insignis* | 25.99334 | -81.57929 |
| *C. insignis* | 25.99334 | -81.57929 |
| *C. insignis* | 26.00286 | -81.57828 |
| *C. insignis* | 27.06456 | -82.27271 |
| *C. insignis* | 27.06456 | -82.27271 |
| *C. insignis* | 27.06468 | -82.26184 |
| *C. insignis* | 27.06468 | -82.26184 |
| *C. insignis* | 27.06215 | -82.28706 |
| *C. insignis* | 27.06215 | -82.28706 |
| *C. insignis* | 27.07085 | -82.28932 |
| *C. insignis* | 27.10428 | -82.31803 |
| *C. insignis* | 27.07890 | -82.28597 |
| *C. insignis* | 27.06906 | -82.28720 |
| *C. insignis* | 27.09649 | -82.31907 |
| *C. insignis* | 27.43069 | -82.31852 |
| *C. insignis* | 27.43069 | -82.31852 |
| *C. insignis* | 27.42931 | -82.32015 |
| *C. insignis* | 27.42931 | -82.32015 |
| *C. insignis* | 27.42931 | -82.32015 |
| *C. insignis* | 27.42931 | -82.32015 |
| *C. insignis* | 27.42931 | -82.32015 |
| *C. insignis* | 27.43069 | -82.31852 |
| *C. insignis* | 27.43069 | -82.31852 |
| *C. insignis* | 27.42931 | -82.32015 |
| *C. insignis* | 27.42931 | -82.32015 |
| *C. insignis* | 27.42931 | -82.32015 |
| *C. insignis* | 27.42931 | -82.32015 |
| *C. insignis* | 27.42931 | -82.32015 |
| *C. insignis* | 26.73991 | -81.70130 |
| *C. insignis* | 26.75442 | -81.69312 |
| *C. insignis* | 26.74048 | -81.71358 |
| *C. insignis* | 26.74492 | -81.71271 |
| *C. insignis* | 26.64417 | -81.70303 |
| *C. insignis* | 26.93650 | -81.32524 |
| *C. insignis* | 26.94363 | -81.31768 |
| *C. insignis* | 26.41081 | -81.15762 |
| *C. insignis* | 26.40954 | -81.15247 |
| *C. insignis* | 26.40806 | -81.14590 |
| *C. insignis* | 26.40791 | -81.12522 |
| *C. insignis* | 26.40595 | -81.13175 |
| *C. insignis* | 26.41172 | -81.12204 |
| *C. insignis* | 26.41065 | -81.12914 |
| *C. insignis* | 26.40155 | -81.13364 |
| *C. insignis* | 26.40286 | -81.13224 |
| *C. insignis* | 26.40426 | -81.13260 |
| *C. insignis* | 26.51274 | -82.09068 |
| *C. insignis* | 26.51079 | -82.09133 |
| *C. insignis* | 26.51070 | -82.09420 |
| *C. insignis* | 26.51068 | -82.09800 |
| *C. insignis* | 26.39001 | -81.00146 |
| *C. insignis* | 26.39051 | -81.00583 |
| *C. insignis* | 26.39087 | -81.00638 |
| *C. insignis* | 26.38171 | -81.02446 |
| *C. insignis* | 26.39504 | -81.04018 |
| *C. insignis* | 26.39917 | -81.04657 |
| *C. insignis* | 26.38998 | -81.03688 |
| *C. insignis* | 26.38469 | -81.03104 |
| *C. insignis* | 26.98901 | -81.95975 |
| *C. insignis* | 26.99887 | -81.96772 |
| *C. insignis* | 26.99689 | -81.96326 |
| *C. insignis* | 26.19969 | -81.65824 |
| *C. insignis* | 26.19934 | -81.67123 |
| *C. insignis* | 26.19352 | -81.66936 |
| *C. insignis* | 27.15239 | -81.75129 |
| *C. insignis* | 27.19410 | -81.74295 |
| *C. insignis* | 27.18701 | -81.74298 |
| *C. insignis* | 27.18043 | -81.74314 |
| *C. insignis* | 27.17988 | -81.75051 |
| *C. insignis* | 26.38230 | -81.75922 |
| *C. insignis* | 26.37564 | -81.75947 |
| *C. insignis* | 26.37839 | -81.76111 |
| *C. insignis* | 26.38764 | -81.76238 |
| *C. insignis* | 26.38544 | -81.75684 |
| *C. insignis* | 26.38219 | -81.76237 |
| *C. insignis* | 26.38266 | -81.76761 |
| *C. insignis* | 26.38626 | -81.77028 |
| *C. insignis* | 27.43069 | -82.31852 |
| *C. insignis* | 27.43069 | -82.31852 |
| *C. insignis* | 27.43069 | -82.31852 |
| *C. insignis* | 27.42931 | -82.32015 |
| *C. insignis* | 27.42931 | -82.32015 |
| *C. insignis* | 27.42931 | -82.32015 |
| *C. insignis* | 27.42931 | -82.32015 |
| *C. insignis* | 27.42931 | -82.32015 |
| *C. insignis* | 27.43069 | -82.31852 |
| *C. insignis* | 27.43069 | -82.31852 |
| *C. insignis* | 27.43069 | -82.31852 |
| *C. insignis* | 27.42931 | -82.32015 |
| *C. insignis* | 27.42931 | -82.32015 |
| *C. insignis* | 27.42931 | -82.32015 |
| *C. insignis* | 27.42931 | -82.32015 |
| *C. insignis* | 27.42931 | -82.32015 |
| *C. insignis* | 26.73010 | -81.64650 |
| *C. insignis* | 26.73140 | -81.64874 |
| *C. insignis* | 27.34597 | -82.36383 |
| *C. insignis* | 26.31923 | -81.61944 |
| *C. insignis* | 26.33228 | -81.61973 |
| *C. insignis* | 27.46506 | -81.52320 |
| *C. insignis* | 27.43069 | -82.31852 |
| *C. insignis* | 27.43069 | -82.31852 |
| *C. insignis* | 27.43069 | -82.31852 |
| *C. insignis* | 27.43069 | -82.31852 |
| *C. insignis* | 27.42931 | -82.32015 |
| *C. insignis* | 27.42931 | -82.32015 |
| *C. insignis* | 27.42931 | -82.32015 |
| *C. insignis* | 27.43069 | -82.31852 |
| *C. insignis* | 27.43069 | -82.31852 |
| *C. insignis* | 27.42931 | -82.32015 |
| *C. insignis* | 27.42931 | -82.32015 |
| *C. insignis* | 27.42931 | -82.32015 |
| *C. insignis* | 27.42931 | -82.32015 |
| *C. insignis* | 27.42931 | -82.32015 |
| *C. insignis* | 28.25253 | -82.42763 |
| *C. insignis* | 28.24923 | -82.42178 |
| *C. insignis* | 28.28500 | -82.41892 |
| *C. insignis* | 28.28300 | -82.41845 |
| *C. insignis* | 26.50850 | -81.28028 |
| *C. insignis* | 26.54023 | -81.29292 |
| *C. insignis* | 26.58061 | -81.30032 |
| *C. insignis* | 26.55598 | -81.29597 |
| *C. insignis* | 26.51841 | -81.28234 |
| *C. insignis* | 26.57277 | -81.37495 |
| *C. insignis* | 26.60872 | -81.35066 |
| *C. insignis* | 25.60061 | -80.39730 |
| *C. insignis* | 25.60314 | -80.39571 |
| *C. insignis* | 25.61007 | -80.40536 |
| *C. insignis* | 26.97206 | -82.28867 |
| *C. insignis* | 26.98617 | -82.27385 |
| *C. insignis* | 26.76677 | -81.84555 |
| *C. insignis* | 26.76614 | -81.84998 |
| *C. insignis* | 26.76823 | -81.84296 |
| *C. insignis* | 26.76734 | -81.84664 |
| *C. insignis* | 26.76611 | -81.84796 |
| *C. insignis* | 26.76713 | -81.85714 |
| *C. insignis* | 26.76793 | -81.85032 |
| *C. insignis* | 26.76663 | -81.85383 |
| *C. insignis* | 27.60746 | -80.76002 |
| *C. insignis* | 27.61295 | -80.75740 |
| *C. insignis* | 27.61900 | -80.75953 |
| *C. insignis* | 27.62304 | -80.76142 |
| *C. insignis* | 27.62901 | -80.76438 |
| *C. insignis* | 28.00325 | -80.75380 |
| *C. insignis* | 28.25259 | -80.81949 |
| *C. insignis* | 28.25039 | -80.82013 |
| *C. insignis* | 28.24793 | -80.82022 |
| *C. insignis* | 28.24706 | -80.81750 |
| *C. insignis* | 28.24715 | -80.81609 |
| *C. insignis* | 28.24856 | -80.80769 |
| *C. insignis* | 28.24936 | -80.80301 |
| *C. insignis* | 28.24958 | -80.80114 |
| *C. insignis* | 28.24646 | -80.82036 |
| *C. insignis* | 28.24414 | -80.82048 |
| *C. insignis* | 27.06968 | -82.04622 |
| *C. insignis* | 27.06841 | -82.04026 |
| *C. insignis* | 27.07499 | -82.03466 |
| *C. insignis* | 27.07595 | -82.05570 |
| *C. insignis* | 27.07597 | -82.05357 |
| *C. insignis* | 27.08340 | -82.05741 |
| *C. insignis* | 27.07598 | -82.05724 |
| *C. insignis* | 27.08030 | -82.02434 |
| *C. insignis* | 27.81450 | -82.16222 |
| *C. insignis* | 27.82227 | -82.16577 |
| *C. insignis* | 26.54873 | -81.46285 |
| *C. insignis* | 26.55221 | -81.46061 |
| *C. insignis* | 26.54845 | -81.46531 |
| *C. insignis* | 26.54342 | -81.46204 |
| *C. insignis* | 26.55127 | -81.46404 |
| *C. insignis* | 27.42931 | -82.32015 |
| *C. insignis* | 27.42931 | -82.32015 |
| *C. insignis* | 27.42931 | -82.32015 |
| *C. insignis* | 27.42931 | -82.32015 |
| *C. insignis* | 27.42931 | -82.32015 |
| *C. insignis* | 26.30121 | -81.79157 |
| *C. insignis* | 26.72631 | -81.59987 |
| *C. insignis* | 26.73913 | -81.59897 |
| *C. insignis* | 26.74908 | -81.52677 |
| *C. insignis* | 26.74908 | -81.52677 |
| *C. insignis* | 26.74908 | -81.52677 |
| *C. insignis* | 26.74908 | -81.52677 |
| *C. insignis* | 26.74908 | -81.52677 |
| *C. insignis* | 26.74908 | -81.52677 |
| *C. insignis* | 26.74908 | -81.52677 |
| *C. insignis* | 26.74908 | -81.52677 |
| *C. insignis* | 26.74908 | -81.52677 |
| *C. insignis* | 26.74908 | -81.52677 |
| *C. insignis* | 26.74908 | -81.52677 |
| *C. insignis* | 26.74908 | -81.52677 |
| *C. insignis* | 26.74908 | -81.52677 |
| *C. insignis* | 26.44033 | -81.52492 |
| *C. insignis* | 26.44630 | -81.51065 |
| *C. insignis* | 27.08504 | -80.20538 |
| *C. insignis* | 27.08869 | -80.21279 |
| *C. insignis* | 27.10864 | -80.21116 |
| *C. insignis* | 27.07511 | -82.32241 |
| *C. insignis* | 27.08046 | -82.32172 |
| *C. insignis* | 27.08531 | -82.33070 |
| *C. insignis* | 27.08746 | -82.33185 |
| *C. insignis* | 26.72993 | -81.85940 |
| *C. insignis* | 26.74556 | -81.85894 |
| *C. insignis* | 26.74797 | -81.86169 |
| *C. insignis* | 26.73356 | -81.86464 |
| *C. insignis* | 26.41334 | -80.09522 |
| *C. insignis* | 26.74908 | -81.52677 |
| *C. insignis* | 26.74908 | -81.52677 |
| *C. insignis* | 26.74908 | -81.52677 |
| *C. insignis* | 26.74908 | -81.52677 |
| *C. insignis* | 26.74908 | -81.52677 |
| *C. insignis* | 26.74908 | -81.52677 |
| *C. insignis* | 27.43069 | -82.31852 |
| *C. insignis* | 27.43069 | -82.31852 |
| *C. insignis* | 27.43069 | -82.31852 |
| *C. insignis* | 27.42931 | -82.32015 |
| *C. insignis* | 27.42931 | -82.32015 |
| *C. insignis* | 27.42931 | -82.32015 |
| *C. insignis* | 27.42931 | -82.32015 |
| *C. insignis* | 27.42931 | -82.32015 |
| *C. insignis* | 27.43069 | -82.31852 |
| *C. insignis* | 27.43069 | -82.31852 |
| *C. insignis* | 27.43069 | -82.31852 |
| *C. insignis* | 27.42931 | -82.32015 |
| *C. insignis* | 27.42931 | -82.32015 |
| *C. insignis* | 27.42931 | -82.32015 |
| *C. insignis* | 27.42931 | -82.32015 |
| *C. insignis* | 27.42931 | -82.32015 |
| *C. insignis* | 26.78311 | -81.17641 |
| *C. insignis* | 26.78301 | -81.17744 |
| *C. insignis* | 28.48516 | -81.10025 |
| *C. insignis* | 28.50165 | -81.12623 |
| *C. insignis* | 26.68995 | -81.60146 |
| *C. insignis* | 26.68440 | -81.60165 |
| *C. insignis* | 26.68646 | -81.59972 |
| *C. insignis* | 26.62523 | -81.78129 |
| *C. insignis* | 26.62675 | -81.78689 |
| *C. insignis* | 26.62634 | -81.78599 |
| *C. insignis* | 26.49426 | -81.17631 |
| *C. insignis* | 26.50184 | -81.18992 |
| *C. insignis* | 26.49946 | -81.19464 |
| *C. insignis* | 26.46313 | -81.20199 |
| *C. insignis* | 26.46857 | -81.18945 |
| *C. insignis* | 26.44265 | -81.18946 |
| *C. insignis* | 27.32799 | -81.84100 |
| *C. insignis* | 27.31814 | -81.83933 |
| *C. insignis* | 27.32576 | -81.84096 |
| *C. insignis* | 27.31937 | -81.84265 |
| *C. insignis* | 27.32942 | -81.84402 |
| *C. insignis* | 26.74908 | -81.52677 |
| *C. insignis* | 26.74908 | -81.52677 |
| *C. insignis* | 26.74908 | -81.52677 |
| *C. insignis* | 26.74908 | -81.52677 |
| *C. insignis* | 26.74908 | -81.52677 |
| *C. insignis* | 26.74908 | -81.52677 |
| *C. insignis* | 26.74908 | -81.52677 |
| *C. insignis* | 27.15177 | -82.10643 |
| *C. insignis* | 27.14366 | -82.10651 |
| *C. insignis* | 27.13738 | -82.10657 |
| *C. insignis* | 27.12950 | -82.10657 |
| *C. insignis* | 27.12105 | -82.10677 |
| *C. insignis* | 27.15227 | -82.09737 |
| *C. insignis* | 27.14873 | -82.09219 |
| *C. insignis* | 27.14300 | -82.08967 |
| *C. insignis* | 27.13769 | -82.09091 |
| *C. insignis* | 27.13866 | -82.08024 |
| *C. insignis* | 27.17760 | -82.11347 |
| *C. insignis* | 27.17115 | -82.10921 |
| *C. insignis* | 27.17038 | -82.10924 |
| *C. insignis* | 27.27674 | -81.96300 |
| *C. insignis* | 27.28129 | -81.97046 |
| *C. insignis* | 27.28150 | -81.96811 |
| *C. insignis* | 27.28181 | -81.96772 |
| *C. insignis* | 26.53917 | -81.68815 |
| *C. insignis* | 26.53322 | -81.70322 |
| *C. insignis* | 26.00015 | -81.57789 |
| *C. insignis* | 26.74908 | -81.52677 |
| *C. insignis* | 26.74908 | -81.52677 |
| *C. insignis* | 26.74908 | -81.52677 |
| *C. insignis* | 26.64339 | -81.72990 |
| *C. insignis* | 26.64286 | -81.72839 |
| *C. insignis* | 26.64741 | -81.73985 |
| *C. insignis* | 26.64614 | -81.74003 |
| *C. insignis* | 27.31267 | -80.54430 |
| *C. insignis* | 27.31299 | -80.54568 |
| *C. insignis* | 27.30898 | -80.54654 |
| *C. insignis* | 27.42931 | -82.32015 |
| *C. insignis* | 27.42931 | -82.32015 |
| *C. insignis* | 27.42931 | -82.32015 |
| *C. insignis* | 27.43069 | -82.31852 |
| *C. insignis* | 27.43069 | -82.31852 |
| *C. insignis* | 27.42931 | -82.32015 |
| *C. insignis* | 27.42931 | -82.32015 |
| *C. insignis* | 27.42931 | -82.32015 |
| *C. insignis* | 27.43069 | -82.31852 |
| *C. insignis* | 27.47563 | -82.35121 |
| *C. insignis* | 26.45135 | -81.83770 |
| *C. insignis* | 26.71514 | -81.18079 |
| *C. insignis* | 26.70972 | -81.17871 |
| *C. insignis* | 27.23128 | -81.87910 |
| *C. insignis* | 27.22772 | -81.87797 |
| *C. insignis* | 27.22503 | -81.88235 |
| *C. insignis* | 27.22422 | -81.87754 |
| *C. insignis* | 26.74908 | -81.52677 |
| *C. insignis* | 26.74811 | -81.52512 |
| *C. insignis* | 26.99666 | -82.18037 |
| *C. insignis* | 29.02813 | -82.32012 |
| *C. insignis* | 29.02980 | -82.32627 |
| *C. insignis* | 29.02984 | -82.33517 |
| *C. insignis* | 29.02951 | -82.34283 |
| *C. insignis* | 29.02410 | -82.34419 |
| *C. insignis* | 29.01745 | -82.34066 |
| *C. insignis* | 29.03448 | -82.35176 |
| *C. insignis* | 27.22251 | -82.29588 |
| *C. insignis* | 26.74908 | -81.52677 |
| *C. insignis* | 26.74908 | -81.52677 |
| *C. insignis* | 26.74908 | -81.52677 |
| *C. insignis* | 26.74908 | -81.52677 |
| *C. insignis* | 26.74908 | -81.52677 |
| *C. insignis* | 26.74908 | -81.52677 |
| *C. insignis* | 28.32258 | -82.57431 |
| *C. insignis* | 28.31854 | -82.57549 |
| *C. insignis* | 28.31613 | -82.58522 |
| *C. insignis* | 28.31609 | -82.57797 |
| *C. insignis* | 28.30472 | -82.56620 |
| *C. insignis* | 28.30089 | -82.56650 |
| *C. insignis* | 26.44615 | -81.55480 |
| *C. insignis* | 26.44144 | -81.55476 |
| *C. insignis* | 26.43684 | -81.56141 |
| *C. insignis* | 26.45174 | -81.54512 |
| *C. insignis* | 26.45168 | -81.54800 |
| *C. insignis* | 26.45167 | -81.55137 |
| *C. insignis* | 26.45154 | -81.55704 |
| *C. insignis* | 26.74908 | -81.52677 |
| *C. insignis* | 26.74908 | -81.52677 |
| *C. insignis* | 28.26718 | -81.98759 |
| *C. insignis* | 28.26362 | -81.97832 |
| *C. insignis* | 28.26705 | -81.99815 |
| *C. insignis* | 28.29741 | -82.00397 |
| *C. insignis* | 28.27365 | -81.97266 |
| *C. insignis* | 27.46152 | -82.21154 |
| *C. insignis* | 27.43069 | -82.31852 |
| *C. insignis* | 27.43069 | -82.31852 |
| *C. insignis* | 27.43069 | -82.31852 |
| *C. insignis* | 26.86810 | -82.02712 |
| *C. insignis* | 27.37414 | -81.42029 |
| *C. insignis* | 27.36795 | -81.43007 |
| *C. insignis* | 27.36791 | -81.41864 |
| *C. insignis* | 26.74908 | -81.52677 |
| *C. insignis* | 26.74908 | -81.52677 |
| *C. insignis* | 26.74908 | -81.52677 |
| *C. insignis* | 26.74908 | -81.52677 |
| *C. insignis* | 25.61726 | -80.30579 |
| *C. insignis* | 26.61597 | -81.42408 |
| *C. insignis* | 26.63851 | -81.42745 |
| *C. insignis* | 26.61700 | -81.40562 |
| *C. insignis* | 26.59709 | -81.40536 |
| *C. insignis* | 29.80450 | -84.78942 |
| *C. insignis* | 29.80438 | -84.78930 |
| *C. insignis* | 29.80419 | -84.79105 |
| *C. insignis* | 29.82583 | -84.89783 |
| *C. insignis* | 30.78457 | -81.96996 |
| *C. insignis* | 30.78476 | -81.96961 |
| *C. insignis* | 30.79388 | -81.93844 |
| *C. insignis* | 30.79411 | -81.93870 |
| *C. insignis* | 30.77931 | -81.96744 |
| *C. insignis* | 30.86937 | -84.94451 |
| *C. insignis* | 29.81916 | -84.90912 |
| *C. insignis* | 30.78462 | -81.96985 |
| *C. insignis* | 30.78478 | -81.96952 |
| *C. insignis* | 30.78516 | -81.96958 |
| *C. insignis* | 30.77967 | -81.96749 |
| *C. insignis* | 30.77900 | -81.96744 |
| *C. insignis* | 30.77850 | -81.96796 |
| *C. insignis* | 27.18628 | -81.19338 |
| *C. insignis* | 27.18628 | -81.19338 |
| *C. insignis* | 27.18628 | -81.19338 |
| *C. insignis* | 27.18628 | -81.19338 |
| *C. insignis* | 27.18628 | -81.19338 |
| *C. insignis* | 27.18628 | -81.19338 |
| *C. insignis* | 27.18628 | -81.19338 |
| *C. insignis* | 27.18628 | -81.19338 |
| *C. insignis* | 27.18628 | -81.19338 |
| *C. insignis* | 27.18628 | -81.19338 |
| *C. insignis* | 27.18628 | -81.19338 |
| *C. insignis* | 27.18628 | -81.19338 |
| *C. insignis* | 27.18628 | -81.19338 |
| *C. insignis* | 27.18628 | -81.19338 |
| *C. insignis* | 27.18628 | -81.19338 |
| *C. insignis* | 27.18628 | -81.19338 |
| *C. insignis* | 27.18628 | -81.19338 |
| *C. insignis* | 27.18628 | -81.19338 |
| *C. insignis* | 27.18628 | -81.19338 |
| *C. insignis* | 27.18628 | -81.19338 |
| *C. insignis* | 27.18628 | -81.19338 |
| *C. insignis* | 27.18628 | -81.19338 |
| *C. insignis* | 27.18628 | -81.19338 |
| *C. insignis* | 27.18628 | -81.19338 |
| *C. insignis* | 27.18628 | -81.19338 |
| *C. insignis* | 27.18628 | -81.19338 |
| *C. insignis* | 27.18628 | -81.19338 |
| *C. insignis* | 27.18628 | -81.19338 |
| *C. insignis* | 27.18628 | -81.19338 |
| *C. insignis* | 27.18628 | -81.19338 |
| *C. insignis* | 27.18628 | -81.19338 |
| *C. insignis* | 27.18628 | -81.19338 |
| *C. insignis* | 27.18628 | -81.19338 |
| *C. insignis* | 27.18628 | -81.19338 |
| *C. insignis* | 27.18628 | -81.19338 |
| *C. insignis* | 27.18628 | -81.19338 |
| *C. insignis* | 27.18628 | -81.19338 |
| *C. insignis* | 27.18628 | -81.19338 |
| *C. insignis* | 27.18628 | -81.19338 |
| *C. insignis* | 27.18628 | -81.19338 |
| *C. insignis* | 27.18628 | -81.19338 |
| *C. insignis* | 27.18628 | -81.19338 |
| *C. insignis* | 27.18628 | -81.19338 |
| *C. insignis* | 27.18628 | -81.19338 |
| *C. insignis* | 27.18628 | -81.19338 |
| *C. insignis* | 27.18628 | -81.19338 |
| *C. insignis* | 27.18628 | -81.19338 |
| *C. insignis* | 27.18628 | -81.19338 |
| *C. insignis* | 27.18628 | -81.19338 |
| *C. insignis* | 27.18628 | -81.19338 |
| *C. insignis* | 27.18628 | -81.19338 |
| *C. insignis* | 27.18628 | -81.19338 |
| *C. insignis* | 27.18628 | -81.19338 |
| *C. insignis* | 27.18628 | -81.19338 |
| *C. insignis* | 27.18628 | -81.19338 |
| *C. insignis* | 27.18628 | -81.19338 |
| *C. insignis* | 27.18628 | -81.19338 |
| *C. insignis* | 27.18628 | -81.19338 |
| *C. insignis* | 27.18628 | -81.19338 |
| *C. insignis* | 27.18628 | -81.19338 |
| *C. insignis* | 26.74853 | -81.52550 |
| *C. insignis* | 26.74853 | -81.52550 |
| *C. insignis* | 26.74853 | -81.52550 |
| *C. insignis* | 26.74853 | -81.52550 |
| *C. insignis* | 26.74853 | -81.52550 |
| *C. insignis* | 26.74853 | -81.52550 |
| *C. insignis* | 26.74853 | -81.52550 |
| *C. insignis* | 26.74853 | -81.52550 |
| *C. insignis* | 26.74853 | -81.52550 |
| *C. insignis* | 26.74853 | -81.52550 |
| *C. insignis* | 26.74853 | -81.52550 |
| *C. insignis* | 26.74853 | -81.52550 |
| *C. insignis* | 26.74853 | -81.52550 |
| *C. insignis* | 26.74853 | -81.52550 |
| *C. insignis* | 26.74853 | -81.52550 |
| *C. insignis* | 26.74853 | -81.52550 |
| *C. insignis* | 26.74853 | -81.52550 |
| *C. insignis* | 26.74853 | -81.52550 |
| *C. insignis* | 26.74853 | -81.52550 |
| *C. insignis* | 26.74853 | -81.52550 |
| *C. insignis* | 26.74853 | -81.52550 |
| *C. insignis* | 26.74853 | -81.52550 |
| *C. insignis* | 26.74853 | -81.52550 |
| *C. insignis* | 26.74853 | -81.52550 |
| *C. insignis* | 26.74853 | -81.52550 |
| *C. insignis* | 26.74853 | -81.52550 |
| *C. insignis* | 26.74853 | -81.52550 |
| *C. insignis* | 26.74853 | -81.52550 |
| *C. insignis* | 26.74853 | -81.52550 |
| *C. insignis* | 26.74853 | -81.52550 |
| *C. insignis* | 26.74853 | -81.52550 |
| *C. insignis* | 26.74853 | -81.52550 |
| *C. insignis* | 26.74853 | -81.52550 |
| *C. insignis* | 29.17891 | -81.97130 |
| *C. insignis* | 29.17891 | -81.97130 |
| *C. insignis* | 29.17891 | -81.97130 |
| *C. insignis* | 29.17891 | -81.97130 |
| *C. insignis* | 29.17891 | -81.97130 |
| *C. insignis* | 29.17891 | -81.97130 |
| *C. insignis* | 29.17891 | -81.97130 |
| *C. insignis* | 29.17891 | -81.97130 |
| *C. insignis* | 29.17891 | -81.97130 |
| *C. insignis* | 29.17891 | -81.97130 |
| *C. insignis* | 29.17891 | -81.97130 |
| *C. insignis* | 29.17891 | -81.97130 |
| *C. insignis* | 29.17891 | -81.97130 |
| *C. insignis* | 29.17891 | -81.97130 |
| *C. insignis* | 29.17891 | -81.97130 |
| *C. insignis* | 29.17891 | -81.97130 |
| *C. insignis* | 29.17891 | -81.97130 |
| *C. insignis* | 29.17891 | -81.97130 |
| *C. insignis* | 28.74024 | -82.03668 |
| *C. insignis* | 28.74024 | -82.03668 |
| *C. insignis* | 28.74024 | -82.03668 |
| *C. insignis* | 28.74024 | -82.03668 |
| *C. insignis* | 28.74024 | -82.03668 |
| *C. insignis* | 28.74024 | -82.03668 |
| *C. insignis* | 28.74024 | -82.03668 |
| *C. insignis* | 27.58889 | -80.47757 |
| *C. insignis* | 27.58050 | -80.48187 |
| *C. insignis* | 27.58889 | -80.47757 |
| *C. insignis* | 27.58050 | -80.48187 |
| *C. insignis* | 27.58050 | -80.48187 |
| *C. insignis* | 27.58889 | -80.47757 |
| *C. insignis* | 27.58050 | -80.48187 |
| *C. insignis* | 27.58889 | -80.47757 |
| *C. insignis* | 27.58828 | -80.40096 |
| *C. insignis* | 27.58828 | -80.40096 |
| *C. insignis* | 27.58050 | -80.48187 |
| *C. insignis* | 27.58050 | -80.48187 |
| *C. insignis* | 27.58649 | -80.37300 |
| *C. insignis* | 27.58649 | -80.37300 |
| *C. insignis* | 27.58050 | -80.48187 |
| *C. insignis* | 27.58649 | -80.37300 |
| *C. insignis* | 27.58649 | -80.37300 |
| *C. insignis* | 27.58889 | -80.47757 |
| *C. insignis* | 27.58889 | -80.47757 |
| *C. insignis* | 27.58050 | -80.48187 |
| *C. insignis* | 27.58050 | -80.48187 |
| *C. insignis* | 27.73665 | -80.42068 |
| *C. insignis* | 27.58050 | -80.48187 |
| *C. insignis* | 27.58649 | -80.37300 |
| *C. insignis* | 27.58050 | -80.48187 |
| *C. insignis* | 27.58889 | -80.47757 |
| *C. insignis* | 27.58050 | -80.48187 |
| *C. insignis* | 27.58649 | -80.37300 |
| *C. insignis* | 27.58889 | -80.47757 |
| *C. insignis* | 27.58889 | -80.47757 |
| *C. insignis* | 27.58828 | -80.40096 |
| *C. insignis* | 27.58649 | -80.37300 |
| *C. insignis* | 27.58050 | -80.48187 |
| *C. insignis* | 27.58889 | -80.47757 |
| *C. insignis* | 27.58050 | -80.48187 |
| *C. insignis* | 27.58889 | -80.47757 |
| *C. insignis* | 27.58050 | -80.48187 |
| *C. insignis* | 27.58656 | -80.36902 |
| *C. insignis* | 27.58656 | -80.36902 |
| *C. insignis* | 27.58649 | -80.37300 |
| *C. insignis* | 27.73665 | -80.42068 |
| *C. insignis* | 30.35810 | -82.91891 |
| *C. insignis* | 30.35810 | -82.91891 |
| *C. insignis* | 30.35810 | -82.91891 |
| *C. insignis* | 30.35810 | -82.91891 |
| *C. insignis* | 30.35810 | -82.91891 |
| *C. insignis* | 30.35810 | -82.91891 |
| *C. insignis* | 30.35810 | -82.91891 |
| *C. insignis* | 30.35810 | -82.91891 |
| *C. insignis* | 30.35810 | -82.91891 |
| *C. insignis* | 30.35810 | -82.91891 |
| *C. insignis* | 30.35810 | -82.91891 |
| *C. insignis* | 30.35810 | -82.91891 |
| *C. insignis* | 30.35810 | -82.91891 |
| *C. insignis* | 30.35810 | -82.91891 |
| *C. insignis* | 30.35810 | -82.91891 |
| *C. insignis* | 30.35810 | -82.91891 |
| *C. insignis* | 30.35810 | -82.91891 |
| *C. insignis* | 30.35810 | -82.91891 |
| *C. insignis* | 30.35810 | -82.91891 |
| *C. insignis* | 30.35810 | -82.91891 |
| *C. insignis* | 30.35810 | -82.91891 |
| *C. insignis* | 30.35810 | -82.91891 |
| *C. insignis* | 30.35810 | -82.91891 |
| *C. insignis* | 30.35810 | -82.91891 |
| *C. insignis* | 30.35810 | -82.91891 |
| *C. insignis* | 30.35810 | -82.91891 |
| *C. insignis* | 30.35810 | -82.91891 |
| *C. insignis* | 30.35810 | -82.91891 |
| *C. insignis* | 30.35810 | -82.91891 |
| *C. insignis* | 30.35810 | -82.91891 |
| *C. insignis* | 30.35810 | -82.91891 |
| *C. insignis* | 30.35810 | -82.91891 |
| *C. insignis* | 30.35810 | -82.91891 |
| *C. insignis* | 30.35810 | -82.91891 |
| *C. insignis* | 30.35810 | -82.91891 |
| *C. insignis* | 30.35810 | -82.91891 |
| *C. insignis* | 30.35810 | -82.91891 |
| *C. insignis* | 30.35810 | -82.91891 |
| *C. insignis* | 30.35810 | -82.91891 |
| *C. insignis* | 30.35810 | -82.91891 |
| *C. insignis* | 30.35810 | -82.91891 |
| *C. insignis* | 30.35810 | -82.91891 |
| *C. insignis* | 30.35810 | -82.91891 |
| *C. insignis* | 30.35810 | -82.91891 |
| *C. insignis* | 30.35810 | -82.91891 |
| *C. insignis* | 30.35810 | -82.91891 |
| *C. insignis* | 30.35810 | -82.91891 |
| *C. insignis* | 30.35810 | -82.91891 |
| *C. insignis* | 30.35810 | -82.91891 |
| *C. insignis* | 30.35810 | -82.91891 |
| *C. insignis* | 30.35810 | -82.91891 |
| *C. insignis* | 30.35810 | -82.91891 |
| *C. insignis* | 30.35810 | -82.91891 |
| *C. insignis* | 30.35810 | -82.91891 |
| *C. insignis* | 28.94360 | -82.01759 |
| *C. insignis* | 28.94360 | -82.01759 |
| *C. insignis* | 28.94360 | -82.01759 |
| *C. insignis* | 28.94360 | -82.01759 |
| *C. insignis* | 28.94360 | -82.01759 |
| *C. insignis* | 28.94360 | -82.01759 |
| *C. insignis* | 28.94360 | -82.01759 |
| *C. insignis* | 28.94360 | -82.01759 |
| *C. insignis* | 28.94360 | -82.01759 |
| *C. insignis* | 28.94360 | -82.01759 |
| *C. insignis* | 28.94360 | -82.01759 |
| *C. insignis* | 28.94360 | -82.01759 |
| *C. insignis* | 30.53801 | -84.52958 |
| *C. insignis* | 30.53801 | -84.52958 |
| *C. insignis* | 30.53801 | -84.52958 |
| *C. insignis* | 30.53801 | -84.52958 |
| *C. insignis* | 30.53801 | -84.52958 |
| *C. insignis* | 30.53801 | -84.52958 |
| *C. insignis* | 30.69742 | -85.17788 |
| *C. insignis* | 30.69742 | -85.17788 |
| *C. insignis* | 30.69742 | -85.17788 |
| *C. insignis* | 30.69742 | -85.17788 |
| *C. insignis* | 30.69742 | -85.17788 |
| *C. insignis* | 30.69742 | -85.17788 |
| *C. insignis* | 30.69742 | -85.17788 |
| *C. insignis* | 30.69742 | -85.17788 |
| *C. insignis* | 30.69742 | -85.17788 |
| *C. insignis* | 30.69742 | -85.17788 |
| *C. insignis* | 30.69742 | -85.17788 |
| *C. insignis* | 30.69742 | -85.17788 |
| *C. insignis* | 30.69742 | -85.17788 |
| *C. insignis* | 30.69742 | -85.17788 |
| *C. insignis* | 30.69742 | -85.17788 |
| *C. insignis* | 30.69742 | -85.17788 |
| *C. insignis* | 30.69742 | -85.17788 |
| *C. insignis* | 30.69742 | -85.17788 |
| *C. insignis* | 30.69742 | -85.17788 |
| *C. insignis* | 30.69742 | -85.17788 |
| *C. insignis* | 30.69742 | -85.17788 |
| *C. insignis* | 30.69742 | -85.17788 |
| *C. insignis* | 30.69742 | -85.17788 |
| *C. insignis* | 30.69742 | -85.17788 |
| *C. insignis* | 30.69742 | -85.17788 |
| *C. insignis* | 30.69742 | -85.17788 |
| *C. insignis* | 30.69742 | -85.17788 |
| *C. insignis* | 27.46566 | -80.32316 |
| *C. insignis* | 27.46566 | -80.32316 |
| *C. stellifer* | 26.51079 | -82.09133 |
| *C. stellifer* | 26.51274 | -82.09068 |
| *C. stellifer* | 26.64339 | -81.72990 |
| *C. stellifer* | 26.68440 | -81.60165 |
| *C. stellifer* | 26.68646 | -81.59972 |
| *C. stellifer* | 26.68995 | -81.60146 |
| *C. stellifer* | 26.71514 | -81.18079 |
| *C. stellifer* | 26.72689 | -81.10924 |
| *C. stellifer* | 26.73277 | -81.64752 |
| *C. stellifer* | 26.73368 | -81.60469 |
| *C. stellifer* | 26.74853 | -81.52550 |
| *C. stellifer* | 26.74908 | -81.52677 |
| *C. stellifer* | 27.07350 | -82.05738 |
| *C. stellifer* | 27.17184 | -82.10942 |
| *C. stellifer* | 27.17760 | -82.11347 |
| *C. stellifer* | 27.18628 | -81.19338 |
| *C. stellifer* | 27.18672 | -82.37286 |
| *C. stellifer* | 27.19800 | -81.91823 |
| *C. stellifer* | 27.22772 | -81.87797 |
| *C. stellifer* | 27.23128 | -81.87910 |
| *C. stellifer* | 27.27465 | -81.96596 |
| *C. stellifer* | 27.27674 | -81.96300 |
| *C. stellifer* | 27.27711 | -81.96866 |
| *C. stellifer* | 27.27891 | -81.96567 |
| *C. stellifer* | 27.28122 | -81.97038 |
| *C. stellifer* | 27.28129 | -81.97046 |
| *C. stellifer* | 27.28181 | -81.96772 |
| *C. stellifer* | 27.28224 | -81.96552 |
| *C. stellifer* | 27.31163 | -80.54585 |
| *C. stellifer* | 27.31299 | -80.54568 |
| *C. stellifer* | 27.31895 | -81.84453 |
| *C. stellifer* | 27.32042 | -81.84583 |
| *C. stellifer* | 27.32193 | -81.83868 |
| *C. stellifer* | 27.32200 | -81.84138 |
| *C. stellifer* | 27.32256 | -81.84041 |
| *C. stellifer* | 27.32365 | -81.84027 |
| *C. stellifer* | 27.32555 | -81.84407 |
| *C. stellifer* | 27.32675 | -81.84876 |
| *C. stellifer* | 27.32799 | -81.84100 |
| *C. stellifer* | 27.32844 | -81.84167 |
| *C. stellifer* | 27.32877 | -81.84965 |
| *C. stellifer* | 27.33068 | -81.84341 |
| *C. stellifer* | 27.36791 | -81.41864 |
| *C. stellifer* | 27.36795 | -81.43007 |
| *C. stellifer* | 27.42791 | -82.32137 |
| *C. stellifer* | 27.42931 | -82.32015 |
| *C. stellifer* | 27.43066 | -82.32156 |
| *C. stellifer* | 27.43069 | -82.31852 |
| *C. stellifer* | 27.46152 | -82.21154 |
| *C. stellifer* | 27.47099 | -81.54221 |
| *C. stellifer* | 27.47183 | -81.53558 |
| *C. stellifer* | 27.47274 | -81.54390 |
| *C. stellifer* | 27.47563 | -82.33632 |
| *C. stellifer* | 27.47569 | -81.54226 |
| *C. stellifer* | 27.47611 | -81.55045 |
| *C. stellifer* | 27.47714 | -82.34015 |
| *C. stellifer* | 27.47810 | -82.33252 |
| *C. stellifer* | 27.47822 | -81.56290 |
| *C. stellifer* | 27.58050 | -80.48187 |
| *C. stellifer* | 27.58649 | -80.37300 |
| *C. stellifer* | 27.58889 | -80.47757 |
| *C. stellifer* | 27.64600 | -82.14224 |
| *C. stellifer* | 27.67615 | -82.35859 |
| *C. stellifer* | 27.67814 | -82.34932 |
| *C. stellifer* | 27.78005 | -81.44835 |
| *C. stellifer* | 27.78344 | -82.14130 |
| *C. stellifer* | 28.00325 | -80.75380 |
| *C. stellifer* | 28.09820 | -81.56525 |
| *C. stellifer* | 28.09843 | -81.56569 |
| *C. stellifer* | 28.09862 | -81.56543 |
| *C. stellifer* | 28.10487 | -81.56303 |
| *C. stellifer* | 28.24079 | -82.42292 |
| *C. stellifer* | 28.25253 | -82.42763 |
| *C. stellifer* | 28.26718 | -81.98759 |
| *C. stellifer* | 28.27365 | -81.97266 |
| *C. stellifer* | 28.29741 | -82.00397 |
| *C. stellifer* | 28.50165 | -81.12623 |
| *C. stellifer* | 28.72707 | -81.48881 |
| *C. stellifer* | 28.74024 | -82.03668 |
| *C. stellifer* | 28.93361 | -82.32768 |
| *C. stellifer* | 28.94360 | -82.01759 |
| *C. stellifer* | 29.08297 | -81.56145 |
| *C. stellifer* | 29.12858 | -81.89667 |
| *C. stellifer* | 29.17891 | -81.97130 |
| *C. stellifer* | 29.47989 | -82.97636 |
| *C. stellifer* | 29.54414 | -82.30103 |
| *C. stellifer* | 29.56460 | -81.55664 |
| *C. stellifer* | 29.57193 | -81.64622 |
| *C. stellifer* | 29.57203 | -81.64816 |
| *C. stellifer* | 29.71386 | -82.45996 |
| *C. stellifer* | 29.75030 | -83.34534 |
| *C. stellifer* | 29.80445 | -84.79052 |
| *C. stellifer* | 29.80471 | -84.78908 |
| *C. stellifer* | 29.82036 | -84.90918 |
| *C. stellifer* | 29.82215 | -84.90948 |
| *C. stellifer* | 29.82573 | -84.89855 |
| *C. stellifer* | 29.82644 | -84.89882 |
| *C. stellifer* | 29.91792 | -82.58566 |
| *C. stellifer* | 29.95308 | -81.64348 |
| *C. stellifer* | 29.95547 | -81.63077 |
| *C. stellifer* | 29.95650 | -81.61984 |
| *C. stellifer* | 29.96128 | -81.65510 |
| *C. stellifer* | 29.96715 | -81.64626 |
| *C. stellifer* | 30.06651 | -81.83130 |
| *C. stellifer* | 30.06939 | -81.83844 |
| *C. stellifer* | 30.08008 | -81.83943 |
| *C. stellifer* | 30.12646 | -81.57549 |
| *C. stellifer* | 30.12975 | -81.57612 |
| *C. stellifer* | 30.24552 | -84.26064 |
| *C. stellifer* | 30.25185 | -84.25799 |
| *C. stellifer* | 30.27361 | -84.29520 |
| *C. stellifer* | 30.33592 | -82.72642 |
| *C. stellifer* | 30.33785 | -82.72157 |
| *C. stellifer* | 30.33931 | -82.69575 |
| *C. stellifer* | 30.34191 | -85.55355 |
| *C. stellifer* | 30.34287 | -85.55390 |
| *C. stellifer* | 30.34355 | -85.55442 |
| *C. stellifer* | 30.34363 | -85.55444 |
| *C. stellifer* | 30.34421 | -85.55479 |
| *C. stellifer* | 30.34427 | -85.55465 |
| *C. stellifer* | 30.35810 | -82.91891 |
| *C. stellifer* | 30.41198 | -85.88335 |
| *C. stellifer* | 30.41335 | -85.88101 |
| *C. stellifer* | 30.46798 | -84.52270 |
| *C. stellifer* | 30.53801 | -84.52958 |
| *C. stellifer* | 30.56960 | -86.92328 |
| *C. stellifer* | 30.56968 | -86.92342 |
| *C. stellifer* | 30.56978 | -86.92343 |
| *C. stellifer* | 30.59256 | -83.71287 |
| *C. stellifer* | 30.66940 | -87.26607 |
| *C. stellifer* | 30.67076 | -87.26421 |
| *C. stellifer* | 30.69742 | -85.17788 |
| *C. stellifer* | 30.71053 | -86.86691 |
| *C. stellifer* | 30.71339 | -86.85814 |
| *C. stellifer* | 30.71371 | -86.86187 |
| *C. stellifer* | 30.71702 | -86.85884 |
| *C. stellifer* | 30.71852 | -86.85872 |
| *C. stellifer* | 30.72046 | -86.85854 |
| *C. stellifer* | 30.72964 | -86.85835 |
| *C. stellifer* | 30.72965 | -86.85877 |
| *C. stellifer* | 30.73055 | -86.80161 |
| *C. stellifer* | 30.73077 | -86.80150 |
| *C. stellifer* | 30.73081 | -86.80158 |
| *C. stellifer* | 30.73324 | -86.85818 |
| *C. stellifer* | 30.77816 | -81.96819 |
| *C. stellifer* | 30.77850 | -81.96796 |
| *C. stellifer* | 30.77900 | -81.96744 |
| *C. stellifer* | 30.77931 | -81.96744 |
| *C. stellifer* | 30.77967 | -81.96749 |
| *C. stellifer* | 30.77975 | -81.96724 |
| *C. stellifer* | 30.77979 | -81.96734 |
| *C. stellifer* | 30.78449 | -81.96980 |
| *C. stellifer* | 30.78457 | -81.96996 |
| *C. stellifer* | 30.78462 | -81.96985 |
| *C. stellifer* | 30.78476 | -81.96961 |
| *C. stellifer* | 30.78478 | -81.96952 |
| *C. stellifer* | 30.78516 | -81.96958 |
| *C. stellifer* | 30.78517 | -81.96980 |
| *C. stellifer* | 30.79388 | -81.93844 |
| *C. stellifer* | 30.79411 | -81.93870 |
| *C. stellifer* | 30.87080 | -84.94492 |
| *C. stellifer* | 30.87091 | -84.94541 |
| *C. stellifer* | 30.90403 | -84.96076 |
| *C. stellifer* | 30.90454 | -84.96045 |
| *C. stellifer* | 30.90471 | -84.96082 |
| *C. stellifer* | 30.90497 | -84.96054 |
| *C. stellifer* | 30.90539 | -84.96130 |
| *C. stellifer* | 30.90541 | -84.96156 |
| *C. stellifer* | 30.90542 | -84.96142 |
| *C. stellifer* | 30.90543 | -84.96159 |
| *C. debilipalpis* | 30.90543 | -84.96159 |
| *C. debilipalpis* | 30.90541 | -84.96156 |
| *C. debilipalpis* | 30.90539 | -84.96130 |
| *C. debilipalpis* | 30.90503 | -84.96081 |
| *C. debilipalpis* | 30.90497 | -84.96054 |
| *C. debilipalpis* | 30.90454 | -84.96045 |
| *C. debilipalpis* | 30.90403 | -84.96076 |
| *C. debilipalpis* | 30.87102 | -84.94579 |
| *C. debilipalpis* | 30.86934 | -84.94422 |
| *C. debilipalpis* | 30.86890 | -84.94400 |
| *C. debilipalpis* | 30.77979 | -81.96734 |
| *C. debilipalpis* | 30.77975 | -81.96724 |
| *C. debilipalpis* | 30.77972 | -81.96741 |
| *C. debilipalpis* | 30.77931 | -81.96744 |
| *C. debilipalpis* | 30.77900 | -81.96744 |
| *C. debilipalpis* | 30.73324 | -86.85818 |
| *C. debilipalpis* | 30.73288 | -86.85799 |
| *C. debilipalpis* | 30.73081 | -86.80158 |
| *C. debilipalpis* | 30.72965 | -86.85877 |
| *C. debilipalpis* | 30.72964 | -86.85835 |
| *C. debilipalpis* | 30.72900 | -86.85863 |
| *C. debilipalpis* | 30.71339 | -86.85814 |
| *C. debilipalpis* | 30.71053 | -86.86691 |
| *C. debilipalpis* | 30.71031 | -86.86597 |
| *C. debilipalpis* | 30.69742 | -85.17788 |
| *C. debilipalpis* | 30.67053 | -87.26488 |
| *C. debilipalpis* | 30.56978 | -86.92343 |
| *C. debilipalpis* | 30.56974 | -86.92370 |
| *C. debilipalpis* | 30.56972 | -86.92360 |
| *C. debilipalpis* | 30.54648 | -87.19537 |
| *C. debilipalpis* | 30.54643 | -87.19534 |
| *C. debilipalpis* | 30.54619 | -87.19552 |
| *C. debilipalpis* | 30.53801 | -84.52958 |
| *C. debilipalpis* | 30.35810 | -82.91891 |
| *C. debilipalpis* | 30.34738 | -84.25442 |
| *C. debilipalpis* | 30.34287 | -85.55390 |
| *C. debilipalpis* | 30.34281 | -85.55389 |
| *C. debilipalpis* | 30.34191 | -85.55355 |
| *C. debilipalpis* | 30.34178 | -85.55325 |
| *C. debilipalpis* | 30.33931 | -82.69575 |
| *C. debilipalpis* | 30.27361 | -84.29520 |
| *C. debilipalpis* | 30.24661 | -84.28658 |
| *C. debilipalpis* | 30.24325 | -84.27989 |
| *C. debilipalpis* | 30.23924 | -84.26429 |
| *C. debilipalpis* | 30.12153 | -81.54704 |
| *C. debilipalpis* | 30.07053 | -81.84415 |
| *C. debilipalpis* | 29.91577 | -82.58129 |
| *C. debilipalpis* | 29.82573 | -84.89855 |
| *C. debilipalpis* | 29.81814 | -84.90901 |
| *C. debilipalpis* | 29.57203 | -81.64816 |
| *C. debilipalpis* | 29.57169 | -81.64969 |
| *C. debilipalpis* | 29.56526 | -81.57637 |
| *C. debilipalpis* | 29.48874 | -82.97222 |
| *C. debilipalpis* | 29.47989 | -82.97636 |
| *C. debilipalpis* | 29.17891 | -81.97130 |
| *C. debilipalpis* | 28.92906 | -82.34201 |
| *C. debilipalpis* | 28.30272 | -82.56425 |
| *C. debilipalpis* | 27.47569 | -81.54226 |
| *C. debilipalpis* | 27.47280 | -81.54404 |
| *C. debilipalpis* | 27.33068 | -81.84341 |
| *C. debilipalpis* | 27.10715 | -81.81635 |
| *C. debilipalpis* | 26.74853 | -81.52550 |
| *C. debilipalpis* | 26.64101 | -81.74160 |
| *C. debilipalpis* | 25.62553 | -80.30543 |
| *C. venustus* | 28.72707 | -81.48881 |
| *C. venustus* | 28.72760 | -81.48177 |
| *C. venustus* | 29.08297 | -81.56145 |
| *C. venustus* | 29.17891 | -81.97130 |
| *C. venustus* | 29.24940 | -82.62420 |
| *C. venustus* | 29.88560 | -84.97515 |
| *C. venustus* | 29.98520 | -81.93613 |
| *C. venustus* | 30.34261 | -84.76087 |
| *C. venustus* | 30.35810 | -82.91891 |
| *C. venustus* | 30.37034 | -84.05515 |
| *C. venustus* | 30.39855 | -85.87015 |
| *C. venustus* | 30.41023 | -85.86813 |
| *C. venustus* | 30.41792 | -85.86378 |
| *C. venustus* | 30.44719 | -84.42899 |
| *C. venustus* | 30.46845 | -84.59064 |
| *C. venustus* | 30.47711 | -84.64844 |
| *C. venustus* | 30.53801 | -84.52958 |
| *C. venustus* | 30.54082 | -84.63767 |
| *C. venustus* | 30.59032 | -85.99873 |
| *C. venustus* | 30.67722 | -86.15391 |
| *C. venustus* | 30.69742 | -85.17788 |
| *C. venustus* | 30.76628 | -86.80505 |
| *C. venustus* | 30.78516 | -81.96958 |
| *C. venustus* | 30.92790 | -86.76302 |
